# The Performance of Niobium-Microalloying Ultra-High-Strength Bridge Cable Steel during Hot Rolling

**DOI:** 10.3390/ma17061259

**Published:** 2024-03-08

**Authors:** Jie Zhou, Zhichao Yu, Jiahui Chen, Sheng Wu, Kaiming Wu, Libo Pan

**Affiliations:** 1 College of Intelligent Manufacturing, Jianghan University, Wuhan 430080, China; 18502703795@163.com (Z.Y.); 15827998857@163.com (J.C.); ws930819@whut.edu.cn (S.W.); pan97181@126.com (L.P.); 2 Hubei Key Laboratory of Advanced Technology for Automotive Components, Wuhan University of Technology, Wuhan 430070, China; 3 The State Key Laboratory of Refractories and Metallurgy, Collaborative Innovation Center for Advanced Steels, Wuhan University of Science and Technology, Wuhan 430080, China; grukang3387@163.com

**Keywords:** niobium-microalloying, bridge cables, hot-rolling process, recrystallization softening, electron backscatter diffraction analysis

## Abstract

This study focuses on exploring the effects of niobium (Nb)-microalloying on the properties of steel for ultra-high-strength bridge cables during hot-rolling processes. We employed a combination of dual-pass compression tests, stress–strain curve analysis, and Electron Backscatter Diffraction (EBSD) techniques to investigate the influence of Nb-microalloying on the static recrystallization behavior and grain size of the steel. The key findings reveal that Nb-microalloying effectively inhibits static recrystallization, particularly at higher temperatures, significantly reducing the volume fraction of recrystallized grains, resulting in a finer grain size and enhanced deformation resistance. Secondly, at a deformation temperature of 975 °C, Nb-containing steel exhibited finer grain sizes compared to Nb-free steel when held for 10 to 50 s; however, the grain size growth accelerated when the hold time exceeded 50 s, likely linked to the increased deformation resistance induced by Nb. Lastly, this research proposes optimal hot-rolling process parameters for new bridge cable steel, recommending specific finishing rolling temperatures and inter-pass times for both Nb-containing and Nb-free steels during the roughing and finishing stages. This study suggests optimal hot-rolling parameters for both Nb-containing and Nb-free steels, providing essential insights for improving hot-rolling and microalloying processes in high-carbon steels for bridge cables.

## 1. Introduction

In bridge engineering, the steel used for bridge cables is a specially designed high-strength material, aimed at meeting the extreme requirements of the load-bearing elements in modern suspension and cable-stayed bridges. The design of this steel encompasses several key performance indicators, including but not limited to high tensile strength, good toughness, and excellent fatigue resistance. These properties enable the bridge cable steel to withstand immense tensile forces without fracturing, ensuring the safety and reliability of the structure [1,2,3]. In bridge design, the use of higher-performance bridge cable steel allows for a reduction in the structure’s self-weight, thereby enabling the possibility of longer-span bridge designs [4].

With the increasing demands for material properties in engineering structures, especially bridges, microalloying technology has become a key method for optimizing the macroscopic properties of steel through microscopic alloy design. Specifically, the addition of Nb (niobium) as a microalloying element has been proven to significantly enhance the mechanical properties of steel. Precise control of Nb not only plays a crucial role in the solid solution strengthening and grain refinement effects of the alloy but also restricts the grain growth through grain boundary pinning mechanisms, thereby maintaining a fine-grained structure in the steel during high-temperature rolling processes [5,6,7,8,9]. Furthermore, grain refinement can improve the uniformity of the material during deformation, reducing the risk of performance degradation caused by temperature changes and stress concentration. Nb also affects the recrystallization behavior of materials, slowing down the recrystallization process, allowing for finer grain sizes at higher deformation temperatures [10,11,12]. Due to these benefits of Nb-microalloying, bridge cable steel in modern bridge engineering increasingly employs this approach [13].

Hot rolling, as one of the critical steps in metal material preparation, plays a decisive role in determining the final microstructure and macroscopic properties of the material. This process, through plastic deformation at high temperatures, enables the material to achieve the desired geometric shape and size. Simultaneously, the internal microstructure of the material undergoes a complex evolution, including recrystallization, grain growth, and phase transformation [5,11,14,15]. Scientific research has shown that fine control of hot-rolling parameters, such as the heating temperature, degree of deformation, deformation speed, and cooling rate, can manipulate the material’s microstructure at the microscopic level, thus achieving targeted performance design at the macroscopic scale [16,17]. For example, in the development of ultra-high-strength steel for bridge cables, hot rolling is not only a means to achieve the desired shape but also a key technological pathway for optimizing material properties and enhancing structural safety.

This study aims to explore the impact of Nb-microalloying on the properties of 2000 MPa grade ultra-high-strength bridge cable steel during hot-rolling processes [6]. It specifically involves simulated dual-pass compression experiments that mimic the hot-rolling process, combined with stress–strain curve analysis and Electron Backscatter Diffraction (EBSD) techniques, to conduct an in-depth analysis of the changes in grain size. This study employs the dual-pass compression test simulation method, which closely approximates the actual rolling process in production. By precisely controlling the deformation parameters, this method reveals the effects of Nb-microalloying on the grain refinement mechanisms during the hot-rolling process.

## 2. Materials and Methods

**Figure 1:**
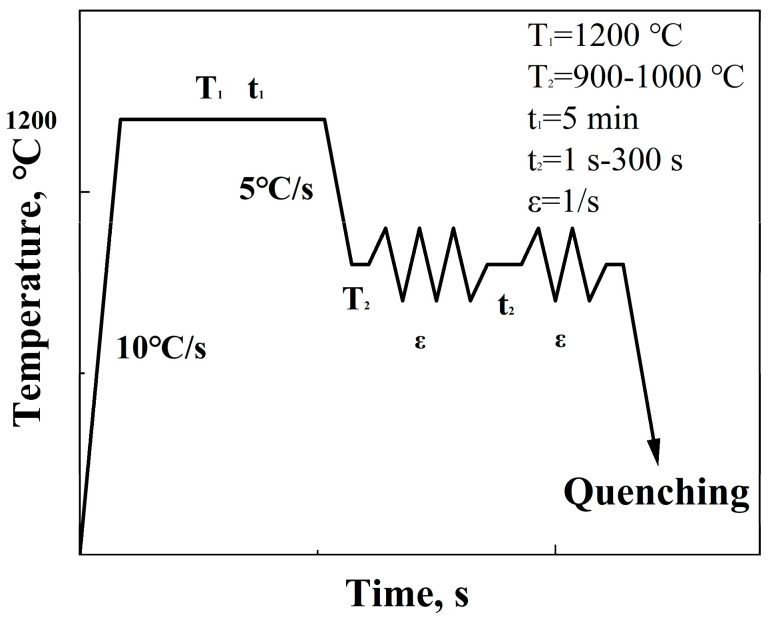
Two–pass compression process schematic diagram.

The experiment utilized high-carbon steel with chemical compositions as shown in Table 1. The materials were refined and produced by the high-speed wire workshop of China WISCO. Sample 1 was Nb-free, while sample 2 contained Nb. A double-pass compression test was conducted on the tested steel using the Gleeble-3500 thermal simulation testing machine. The heat treatment schematic diagram is shown in Figure 1. Samples were first processed by wire-cutting into cylindrical specimens with a diameter of 8 mm and a height of 12 mm. The specimens were rapidly heated to a target temperature of 1200 °C (with a heating rate of 10 °C/s) and held at this temperature for 5 min to homogenize their microstructure. Subsequently, the samples were cooled to different deformation temperatures within the range of 900 °C to 1000 °C (at five temperature points) at a cooling rate of 5 °C/s, for the first compression (true strain of 0.4, strain rate of 1 s^−1^). After the first deformation, they were held at the specified deformation temperature for varying times (ranging from 300 s to 1 s) to simulate different cooling and dwelling conditions, followed by a second compression (with the same true strain and strain rate as the first) and rapid quenching to fix the microstructure. Stress–strain data were collected in real time via the load and displacement sensors on the experimental machine.

Following the recrystallization experiments, the specimens were first cut open along the axial direction, and they then underwent standard metallographic sample preparation procedures, including grinding and polishing until the surface was free of visible scratches. The sample surfaces were subsequently subjected to etching treatment. EBSD scanning was performed using pre-set parameters (acceleration voltage of 20 kV, scan step size of 300 nm) to ensure high-quality diffraction patterns were obtained. After data collection, specialized EBSD software was used for the post-processing, such as noise reduction, grain boundary identification, and grain size measurement, as well as analysis of the crystal orientation distribution. This provided an in-depth understanding of the microstructural characteristics of the materials.

## 3. Experimental Results

### 3.1. Volume Fraction of Static Recrystallization

**Figure 2:**
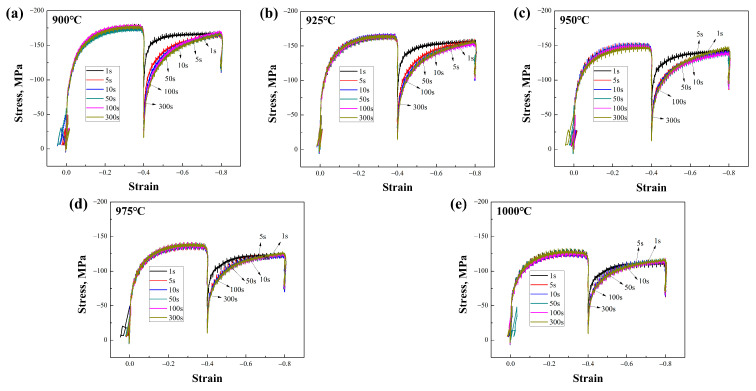
Stress–strain curves of Nb-free steel at (**a**–**e**) 900–1000 °C deformation temperatures and at 1–300 s intervals.

**Figure 3:**
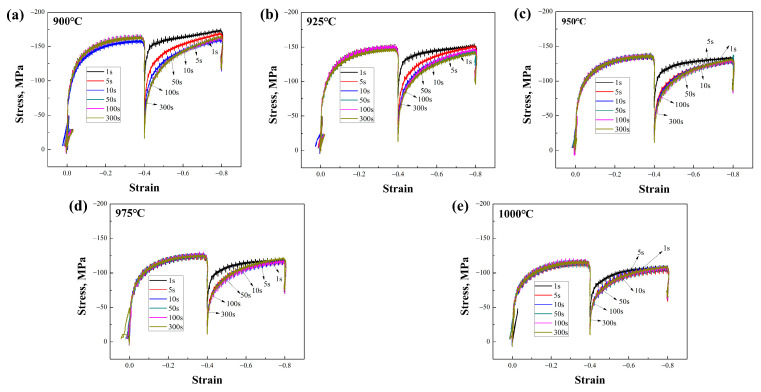
Stress–strain curves of Nb-bearing steel at (**a**–**e**) 900–1000 °C deformation temperatures and at 1–300 s intervals.

During the dual-pass compression experiments at different deformation temperatures and time intervals on the Nb-free and Nb-bearing steels, the collected stress–strain data revealed differences in the static recrystallization behavior of the two materials, as shown in Figure 2 and Figure 3. It can be observed that the deformation resistance of Nb-microalloyed steel is significantly higher than that of Nb-free steel. The stress–strain curves of both can be used to read parameters such as the peak stress during each deformation pass for subsequent calculations. For the quantification of the static softening rate, this study employed a compensation method for the calculation [18]. This method selects the 0.2% plastic deformation yield point as the benchmark, as data collection at this point is convenient and the error is relatively small, with precision superior to the 2% yield stress point. The calculation formula is as follows:

(1) $$R=\left(\sigma_{m}-\sigma_{2}\right)/\left(\sigma_{m}-\sigma_{1}\right)$$

In this formula, $\sigma_{m}$ represents the peak stress at the end of the first pass deformation; $\sigma_{2}$ and $\sigma_{1}$ are the yield stresses for the first and second pass, respectively, where the yield stress refers to the flow stress at 2% strain. The austenite deformation softening rate calculated through the 2% compensation method includes both the contributions of static recovery and the effects of static recrystallization. Based on the understanding that a softening rate of 0.2 marks the onset of recrystallization, the volume fraction of static recrystallization $X_{SRX}$ can be further calculated [19]. The calculation formula is:

(2) $$X_{SRX}=\left(R-0.2\right)/\left(1-0.2\right)=\left(R-0.2\right)/0.8$$

**Figure 4:**
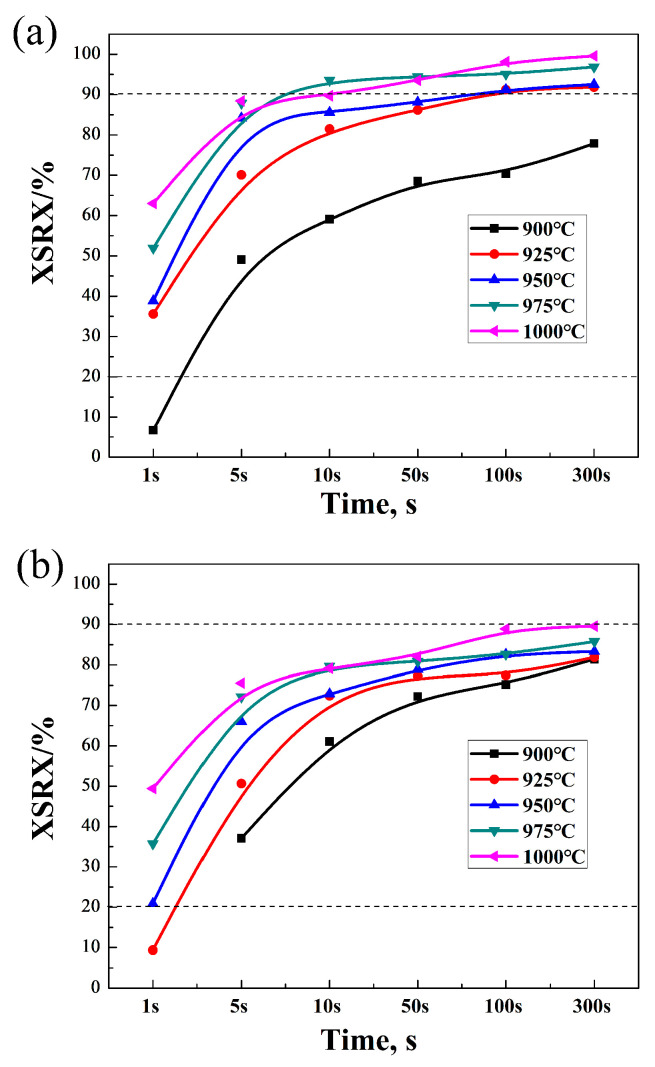
Tested steel static recrystallization softening rate curve: (**a**) Nb-free steel and (**b**) Nb-bearing steel.

Based on the aforementioned method, the static softening rates and volume fractions for both types of steels were calculated and summarized in Table 2. From these data, graphs depicting the relationship between the volume fraction of static recrystallization, deformation temperature, and dwelling time were constructed (Figure 4). The graphs indicate that when the volume fraction of static recrystallization exceeds 90%, complete static recrystallization is considered to have occurred; when the volume fraction is below 20%, static recrystallization is considered not to have occurred [19]. Furthermore, the addition of the microalloying element Nb significantly reduced the volume fraction of static recrystallization in the new experimental steel under the same experimental conditions. This suggests that microalloying with Nb has a significant impact on static recrystallization.

### 3.2. Microstructural Characterization of Static Recrystallization

**Figure 5:**
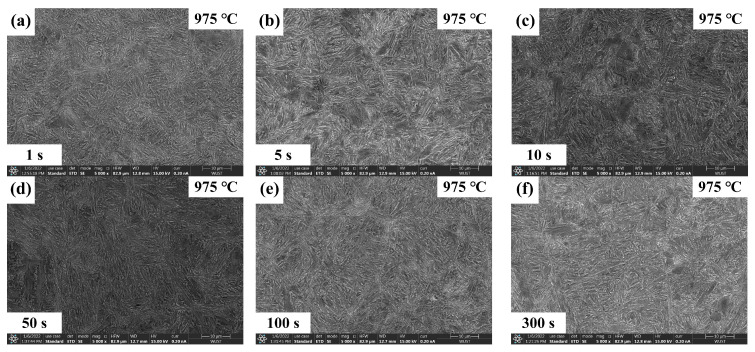
Microstructure of Nb-free steel at 975 °C deformation temperature and (**a**–**f**) 1–300 s residence time.

**Figure 6:**
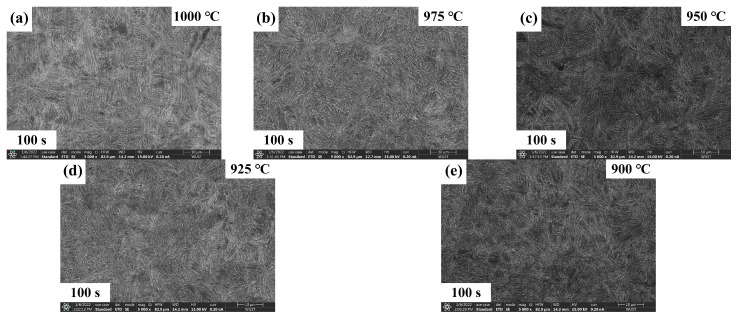
Microstructure of Nb-free steel at (**a**–**e**) 900–1000 °C deformation temperature and 100 s residence time.

**Figure 7:**
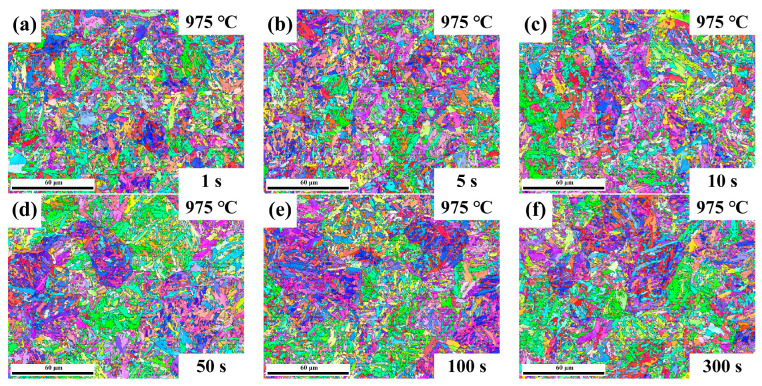
IPF of Nb-free steel at 975 °C deformation temperature and (**a**–**f**) 1–300 s residence time.

**Figure 8:**
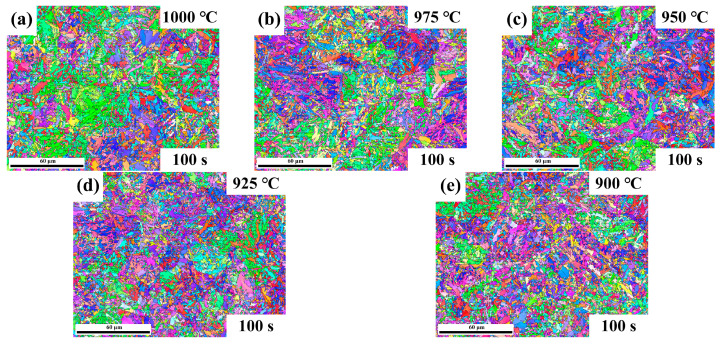
IPF of Nb-free steel at (**a**–**e**) 1000–900 °C deformation temperature and 100 s residence time.

**Figure 9:**
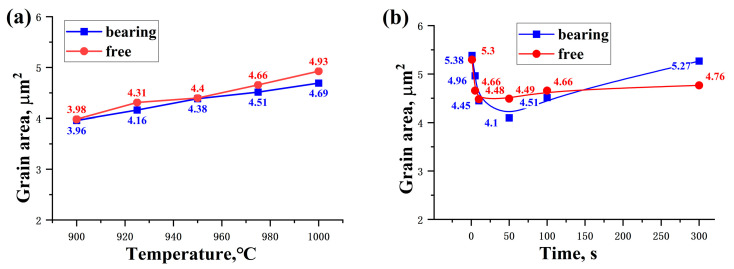
(**a**) Line graph of the average grain area for both types of steels at different deformation temperatures (with a 100 s dwelling time). (**b**) Curve graph of the average grain area for both types of steels at different intermediate dwelling times (at a deformation temperature of 975 °C).

The quenched microstructures of Nb-free steel after static recrystallization are illustrated in Figure 5 and Figure 6. Here, typical experimental conditions at 975 °C for intermediate dwelling times of 1 s to 300 s and deformation temperatures from 900 °C to 1000 °C for a 100 s dwelling time were selected. Both types of experimental steels obtained a substantial amount of martensitic structure after dual-pass rolling and quenching. EBSD analysis was performed for both steels under the same conditions, as shown in Figure 7 and Figure 8. Due to the small difference in the heat treatment temperatures, the difference in the average grain area and size is limited. Considering the experimental error, the grain size data under typical experimental parameters are shown in Figure 9.

The pearlite colony sizes and pearlite colony boundaries of the samples’ varied size angles are displayed in Figure 7a. Figure 7b shows the high- and low-angle grain boundary (H/LAGB) misorientation of the sample, and the statistics are determined with 15° as the boundary.

## 4. Discussion

### 4.1. The Impact of Niobium-Microalloying on Static Recrystallization

The dual-pass compression tests were primarily conducted to study the impact of Nb-microalloying on high-carbon steel during hot-rolling processes, especially regarding static recrystallization [17,20]. Table 2 presents the data for the softening fraction (R/%) and recrystallization volume fraction (X_SRX_/%) of the two types of steels under various deformation temperatures (900 °C to 1000 °C) and intermediate dwelling times (1 s to 300 s). Typically, a higher softening fraction indicates more significant microstructural changes in the material, signifying the extent of the microstructural alterations (such as the dislocation rearrangement and grain restructuring) induced by deformation during hot rolling [21]. For both types of steels, the softening fraction and recrystallization volume fraction generally increase with higher deformation temperatures and longer intermediate dwelling times. This suggests that under higher temperature and longer duration conditions, the material is more prone to recrystallization, leading to more pronounced softening. Comparing Nb-bearing with Nb-free steel under the same experimental conditions, the Nb-bearing steel generally shows lower softening fractions and recrystallization volume fractions. This indicates that the addition of Nb inhibits the recrystallization process, slowing down the softening of the material. This could be due to the formation of niobium carbides, which act as pinning agents at the grain boundaries, inhibiting grain growth and recrystallization [22]. The recrystallization proportion fractions are also more visually evident in Figure 4.

The addition of Nb leads to a significant reduction in the grain size, as well as to changes in the grain morphology and distribution. The microstructure of the samples was analyzed in detail using EBSD technology. However, in this experiment, the factors affecting the grain size included not only the temperature but also the intermediate dwelling time. When the intermediate dwelling time is sufficiently long, and the volume fraction of static recrystallization exceeds 90%, complete static recrystallization is considered to have occurred. In such cases, the expected grain size is smaller because the recrystallization process typically results in the formation of new grains, thereby refining the grain structure. Conversely, when the dwelling time is short and the volume fraction of static recrystallization is below 20%, static recrystallization is considered not to have occurred, and the grain size may be larger due to insufficient new grain formation to replace the original deformed grains. A recrystallization volume fraction between these two extremes is referred to as mixed crystal, a phenomenon that improves with an extended holding time. Figure 9a indicates the average grain area at different deformation temperatures, showing the changes in the average grain area for both types of steels within the 900 °C to 1000 °C deformation temperature range, under a 100 s intermediate dwelling time. Typically, the grain area increases with a rising deformation temperature, as higher temperatures promote grain growth [23]. Additionally, the Nb-bearing steel exhibits a generally smaller average grain area across the entire temperature range compared to the Nb-free steel, indicating that Nb addition effectively inhibits grain growth, maintaining smaller grain sizes even at higher temperatures. Figure 9b shows the average grain area at different intermediate dwelling times. Under longer dwelling times, the grains have more time to grow, thus increasing the average grain area. Conversely, under shorter dwelling times, a mixed crystal state or a lower proportion of recrystallization results in a larger final average grain area. Comparing the two materials, it is evident that Nb-bearing steel can achieve the smallest average grain area of 4.1 μm^2^ under optimal processing parameters, a notable improvement over the 4.48 μm^2^ of Nb-free steel. Although the grain size of Nb-bearing steel increases faster with extended dwelling times, this is mainly due to the increased deformation resistance of Nb-bearing steel. Comparing the dual-pass compression curves in Figure 2 and Figure 3, the stress–strain curves of Nb-bearing steel exhibit higher yield strength and deformation resistance. In hot-rolling or heat-treatment processes, the material’s resistance to deformation is closely related to changes in its microstructure. Nb-bearing steel, due to its higher resistance to deformation, may undergo more deformation work during the dual-pass compression process [12,24]. This deformation work can induce grain boundary movement, accelerating grain restructuring and growth [25].

### 4.2. Analysis of Thermal Deformation Process Parameters of Bridge Cable Steel

The dual-pass compression static recrystallization experiments simulated recrystallization-controlled rolling, involving heating the steel to the austenitization temperature, followed by plastic deformation. During each pass of deformation or between the two passes, dynamic and static recrystallization occurred, completing the recrystallization process. Repeated rolling and recrystallization refined the austenite grains, setting the stage for the formation of fine grains after phase transformation. To prevent the growth of recrystallized austenite grains, it is crucial to strictly control the reduction in the final passes, rolling temperature, and interval time between passes. However, the work hardening of austenite during thermal deformation cannot be completely eliminated, leading to instability in the microstructure. Consequently, the structure after deformation undergoes changes due to static softening at high temperatures. The static softening process, influenced by the amount of hot deformation, can be divided into three stages: static recovery, static recrystallization, and metadynamic recrystallization. Static and metadynamic recrystallization are the main mechanisms of static softening after deformation, determining the degree of softening during the intervals in multi-pass hot rolling. Figure 4 shows that under the same interval, the volume fraction of static recrystallization increases with the deformation temperature. The relationship with the dwelling time shows a rapid increase in the volume fraction of static recrystallization within 1–10 s, followed by a slower increase. This is mainly because the static recrystallization process, including nucleation and growth, is a thermally activated process; the higher the deformation temperature, the greater the nucleation rate of static recrystallization. Grain growth, essentially a grain boundary migration process, requires atomic diffusion, which accelerates with higher deformation temperatures, increasing the boundary migration rates, thus increasing the static recrystallization volume fraction. Related research [26,27] utilized multivariate nonlinear regression analysis to determine the coefficients and establish a model describing the static and dynamic recrystallization of steel microstructures. The study examined the influence of the initial grain size, deformation temperature, strain, and strain rate on the austenite recrystallization volume fraction and grain size. It found that a larger recrystallization volume fraction reduces the grain size during deformation. At the same time, the static recrystallization volume fraction increases with an increasing deformation temperature, strain, strain rate, and decreasing initial grain size. The recrystallization volume fractions calculated in this experiment are consistent with the experimentally measured grain sizes. Figure 4 shows that the recrystallization volume fraction of the experimental steel trends upward with an increasing deformation temperature or interval time between passes. Static recrystallization, including nucleation and growth, increases with extended interval times due to the high dislocation energy in the matrix continuously generating new nucleation sites [28,29]. Higher deformation temperatures cause austenite grains to grow continuously, thus increasing the static recrystallization softening rate and volume fraction. Typically, a static recrystallization softening rate of 90% or more indicates the completion of the recrystallization process. At a deformation temperature of 1000 °C and an interval of 5 s, both Nb-free and Nb-bearing steels achieve a recrystallization volume fraction of over 70%. However, for Nb-bearing steel to reach a 90% recrystallization volume fraction, an interval of over 100 s is needed. It is noteworthy that a recrystallization fraction of 80% or less leads to the mixed crystal phenomenon, indicating that to achieve full recrystallization of the experimental steel, the hot-rolling conditions for Nb-free steel should be above 975 °C with an interval time of more than 10 s, while Nb-bearing steel requires an interval time of over 100 s. Under these conditions, the smallest grain size after two-pass rolling is obtained. Overall, the impact of niobium-microalloying on static recrystallization in hot-rolling processes highlights its potential in refining grains and enhancing material properties. This effect not only helps improve the mechanical properties of materials but is also significant for optimizing material processing and enhancing the performance of the final products in industrial applications.

## 5. Conclusions

This study delves into the impact of niobium-microalloying on the properties of steel used for ultra-high-strength bridge cables during hot-rolling processes. Utilizing dual-pass compression tests, stress–strain curve analysis, and Electron Backscatter Diffraction techniques, we have uncovered significant effects of Nb-microalloying on the static recrystallization behavior and grain size. The key findings are as follows:

1. The experimental results clearly demonstrate that Nb-microalloying can effectively inhibit static recrystallization and promote grain refinement. The addition of Nb significantly reduces the volume fraction of static recrystallization, leading to finer grain sizes and higher deformation resistance.
2. This study also found that under a deformation temperature of 975 °C, Nb-microalloyed high-carbon steel achieves finer grain sizes than Nb-free steel when held for 10–50 s. However, the rate of grain size growth accelerates after an intermediate dwelling time of over 50 s, related to the increased deformation resistance caused by Nb.
3. The optimal hot-rolling conditions for new bridge cable steel are as follows: For Nb-free steel, the finishing temperature in the roughing stage should be ≥1000 °C, and the inter-pass time ≥ 10 s; the starting temperature in the finishing stage should be ≤900 °C, and the inter-pass time ≤ 5 s. For Nb-bearing steel, the finishing temperature in the roughing stage should be ≥1000 °C, and the inter-pass time ≥ 100 s; the starting temperature in the finishing stage should be ≤925 °C, and the inter-pass time ≤ 5 s.

## Figures and Tables

**Table 1:** Chemical composition of the test steel (mass fraction, %).

	Samples	C	Si	Mn	Cr	Nb	P + S	Fe
1	Nb-free	0.98	0.98	0.49	0.35	-	<0.003	Bal
2	Nb-bearing	0.98	0.99	0.48	0.35	0.025	<0.003	Bal

**Table 2:** Softening fraction and recrystallization integral fraction of the tested steel.

Temperature		900 °C		925 °C		950 °C		975 °C		1000 °C	
	Time	R/%	X_SRX_/%	R/%	X_SRX_/%	R/%	X_SRX_/%	R/%	X_SRX_/%	R/%	X_SRX_/%
1	1 s	25.34	6.68	48.46	35.57	51.07	38.86	61.53	51.91	70.38	63.00
	5 s	59.25	49.07	78.46	70.08	87.36	84.20	90.29	87.86	90.74	88.42
	10 s	67.26	59.08	85.17	81.46	88.60	85.57	94.82	93.52	91.75	89.69
	50 s	74.81	68.52	88.95	86.18	90.50	88.12	95.54	94.43	94.82	93.53
	100 s	76.26	70.33	92.98	91.22	93.00	91.25	96.06	95.08	98.50	98.13
	300 s	82.28	77.86	93.46	91.83	94.03	92.54	97.49	96.86	99.67	99.59
2	1 s	15.86	--	27.51	9.39	36.73	20.91	48.55	35.69	59.55	49.43
	5 s	49.66	37.08	60.50	50.63	72.85	66.06	77.72	72.15	80.43	75.53
	10 s	68.87	61.09	77.90	72.38	78.30	72.88	83.76	79.67	83.36	79.20
	50 s	77.79	72.23	81.83	77.29	83.16	78.94	84.87	80.87	85.67	82.08
	100 s	80.13	75.16	81.95	77.44	86.09	82.61	86.11	82.64	91.13	88.92
	300 s	85.13	81.41	85.51	81.89	86.67	83.34	88.63	85.78	91.66	89.57

## Data Availability

Data are contained within the article.
